# Editorial for the Special Issue on Advanced Manufacturing Technology and Systems, 2nd Edition

**DOI:** 10.3390/mi14122243

**Published:** 2023-12-15

**Authors:** Youqiang Xing, Xiuqiang Hao, Duanzhi Duan

**Affiliations:** 1 School of Mechanical Engineering, Southeast University, Nanjing 211189, China; 2 College of Mechanical and Electrical Engineering, Nanjing University of Aeronautics and Astronautics, Nanjing 210016, China; 3 School of Mechanical Engineering, Xi’an Jiaotong University, Xi’an 710049, China

Advanced manufacturing technology and systems (AMTS) combine the principles of mechanical engineering with innovative design to create products and processes that are better, faster, and more precise [1]. The core of AMTS is the design, fabrication, and application of original and effective solutions related to manufacturing machines, process integration, and systems to keep pace with the dynamic needs of today’s ever-evolving industries [2]. AMTS covers a broad scope encompassing automation technology, robots, flexible manufacturing systems, virtual manufacturing, intelligent manufacturing, as well as theoretical study and metrology for promoting manufacturing development [3,4]. This Special Issue presents the latest development in AMTS.

This Special Issue contains 32 original papers, including 2 reviews, related to AMTS. Specifically, many research fields are covered: manufacturing technology (14 papers), structure design (6 papers), system optimization (5 papers), and precision measurement (5 papers). These studies are briefly summarized as follows.

Manufacturing technology: Fan et al. (Contribution 4) have investigated the performance of Ti1-xAlxN-coated tools in milling Ti-6Al-4V alloy, and the form and mechanism of the wear of the Ti1-xAlxN-coated tools were studied. An asymmetric arc hypothesis of a wire bow model has been proposed for diamond wire sawing (Contribution 5). The Bosch, pseudo-Bosch, and cryogenic etching processes have been investigated to fabricate silicon deep trenches with vertical and smooth sidewalls (Contribution 8). The influences of trajectory on the characteristics of low-carbon steel samples generated by the wire arc additive manufacturing (WAAM) technique have been explored (Contribution 9). The impact of the variations in the coating thickness of nano-crystalline diamond on the cutting tool abrasion and quality of a machined surface have been studied (Contribution 11). Wang et al. (Contribution 13) have studied the coaxial waterjet-assisted laser scanning machining of a nickel-based special alloy. The discharge phenomena in the electrochemical discharge machining process has also been investigated (Contribution 16). The micro-milling of Inconel 600 super alloy has been achieved and the effects of the key input parameters on the response parameters under various cooling conditions have been analyzed (Contribution 17). A vibration-assisted diamond wire sawing method has been proposed (Contribution 22). Numerically iterative models have been employed to study two processes (laser heating and energetic plasma plume expansion) involved in the pulsed laser deposition of an Y_3_Fe_5_O_12_ target (Contribution 23). The effect of a punch surface microtexture on the microextrudability of AA6063 micro backward extrusion has been investigated (Contribution 24). The effect of tool coatings on the machining properties of compacted graphite iron has been investigated (Contribution 25). The surface integrity of solar cell silicon wafers sliced by an electrochemical multi-wire saw have been studied (Contribution 26). Nano steps with heights of 1, 2, 3, and 4 nm have been fabricated with good morphology using atomic layer deposition (ALD) combined with wet etching (Contribution 27).

Structure design: Ding et al. (Contribution 1) have designed a prototype structure to meet the requirements of wafer surface and edge defect inspection. Based on the first-principles method, TiAlSiN/WC-Co interface models with graphene doped into the matrix, coating, and the coating/matrix have been constructed (Contribution 15). A longitudinal–torsional transducer has been developed for the ultrasonic vibration-assisted milling (UVAM) of a honeycomb aramid material (Contribution 19). Micro-alternators with two different housing structures—an uncoated shell and a shell coated with an iron-based amorphous alloy soft magnetic material—have been studied (Contribution 21). Micro-dimples have been fabricated on the surface of WC/Co cemented carbide disks by laser, on which dry friction tests have been carried out (Contribution 28). A numerical control shrink-fit holder clamping fatigue test device has been manufactured, and the automatic clamping of the shrink-fit holder has been executed (Contribution 29).

System optimization: An eight-degrees-of-freedom redundant manipulator with one linear and seven rotational joints has been designed to assist with curvature machining (Contribution 7). A robotic compliance control strategy of contact force for ultrasonic surface strengthening has been proposed to satisfy the automatic ultrasonic strengthening requirements of an aviation blade surface (Contribution 10). To address the problem of the low overall machining efficiency of freeform surfaces and the difficulty in ensuring machining quality, a MATLAB-based freeform surface division method has been proposed (Contribution 18). A logic-judgment-based mold breakout prediction system has been developed for a continuous casting machine (Contribution 20). A robust Kalman filtering problem for multisensor time-varying systems with uncertainties of noise variances has been addressed (Contribution 30).

Precision measurement: Wang et al. (Contribution 2) have developed integrated periodic structure reference materials with an expanded measurement range through a combined photolithography and inductively coupled plasma (ICP) etching process. The in situ measurement and reconstruction of the cylindrical shape of a high-precision mandrel by means of a three-point method has been proposed (Contribution 3). A new detection method for wafer surface defects based on background subtraction and Faster R-CNN has been proposed (Contribution 6). A field-effect transistor (FET) based on an MoS_2_/C60 composite nanolayer as the channel material has been fabricated, enabling an ultralow detection limit of miRNA (Contribution 12). To overcome the difficulty of the traditional vibration measurement method, a three-dimensional (3D) measurement method of a micro-milling tool has been proposed based on multi-fiber array coding using the online measurement of the micro-milling tool’s multi-dimensional vibration (Contribution 14). 

Finally, the research progress in abrasive water jet processing technology and visual measurement methods for metal vaporization processes in laser powder bed fusion has been reviewed.

## References

[1] Kuljanic E. (2014). Advanced Manufacturing Systems and Technology.

[2] Chan F.T.S., Chan M.H., Lau H., Ip R.W.L. (2001). Investment Appraisal Techniques for Advanced Manufacturing Technology (AMT): A Literature Review. Integr. Manuf. Syst..

[3] Wang B., Tao F., Fang X., Liu C., Liu Y., Freiheit T. (2021). Smart Manufacturing and Intelligent Manufacturing: A Comparative Review. Engineering.

[4] Weckenborg C., Schumacher P., Thies C., Spengler T.S. (2023). Flexibility in Manufacturing System Design: A Review of Recent Approaches from Operations Research. Eur. J. Oper. Res..

